# ArASL: Arabic Alphabets Sign Language Dataset

**DOI:** 10.1016/j.dib.2019.103777

**Published:** 2019-02-23

**Authors:** Ghazanfar Latif, Nazeeruddin Mohammad, Jaafar Alghazo, Roaa AlKhalaf, Rawan AlKhalaf

**Affiliations:** a College of Computer Engineering and Sciences, Prince Mohammad Bin Fahd University, Al Khobar, Saudi Arabia; b Faculty of Computer Science and Information Technology, Universiti Malaysia Sarawak, Kota Samarahan, Malaysia

## Abstract

A fully-labelled dataset of Arabic Sign Language (ArSL) images is developed for research related to sign language recognition. The dataset will provide researcher the opportunity to investigate and develop automated systems for the deaf and hard of hearing people using machine learning, computer vision and deep learning algorithms. The contribution is a large fully-labelled dataset for Arabic Sign Language (ArSL) which is made publically available and free for all researchers. The dataset which is named ArSL2018 consists of 54,049 images for the 32 Arabic sign language sign and alphabets collected from 40 participants in different age groups. Different dimensions and different variations were present in images which can be cleared using pre-processing techniques to remove noise, center the image, etc. The dataset is made available publicly at https://data.mendeley.com/datasets/y7pckrw6z2/1.

**Table:** Specifications table

Subject area	*Computer Science, Machine Learning, Computer Vision, Deep Learning*
More specific subject area	*Sign Language Recognition for the deaf and hard of hearing*
Type of data	*Images (64×64 pixels JPG format)*
How data was acquired	*Smart Camera (iPhone 6S) used to capture Images.*
Data format	*Labelled Grayscale Images*
Experimental factors	*Preprocessing to resize and grayscale conversion*
Experimental features	*None*
Data source location	*Al Khobar, Eastern Province, Saudi Arabia*
Data accessibility	*The Dataset is made accessible at**https://data.mendeley.com/datasets/y7pckrw6z2/1**[1]**and it is free and publicly available for any research, academic and educational purposes.*
Related research article	*The accuracy was stated in the paper and could serve as a benchmark for research to increase recognition accuracy. The modified version of the paper for journal is already accepted (in press)**[2]**.*

**Table:** 

**Value of the data** • The current trend of machine learning and deep learning in developing applications helpful in our daily lives such as fingerprint or face recognition and other application in fields such as healthcare, assistive technology, and others. The main core of these applications is image pre-processing, classification and recognition to automate tasks usually done by humans. The ArSL2018 dataset is a valuable resource for researchers in the machine learning and deep learning community for development of assistive technology applications for persons with disability. • The ArSL2018 dataset collected in Al Khobar, Saudi Arabia is a collection of 54,000 images of the 32 Arabic Sign Language Signs and Alphabet. • The ArSL2018 is a comprehensive Arabic Sign Language Image repository fully-labelled for purposes of classification and recognition, and for the purpose of applications automating the recognition of sign language for Arabic deaf and hard of hearing individuals. • The ArSL2018 dataset would assist researchers and allow for faster application development, and faster prototyping of different applications and devices in the assistive technology field. • The ArSL2018 is a base for the research community to build on this dataset to produce a dataset with more image variations.

## 1 Data

The ArSL2018 is a new comprehensive fully labelled dataset of Arabic Sign Language images launched in Prince Mohammad Bin Fahd University, Al Khobar, Saudi Arabia to be made available for researchers in the field of Machine Learning and Deep Learning. It is useful for application and device development in the assistive technology field for the benefit of the deaf and hard of hearing individuals. Examples of related datasets can be found in Refs. [3], [4], [5]. The ArSL2018 dataset is unique in the sense that it is the first large comprehensive dataset for Arabic Language Sign Language according to the author(s) knowledge. There is a large potential for this dataset to be used by researchers to both increase accuracies of classification and recognition and for development of prototypes useful for the deaf community.

**Fig. 1:**
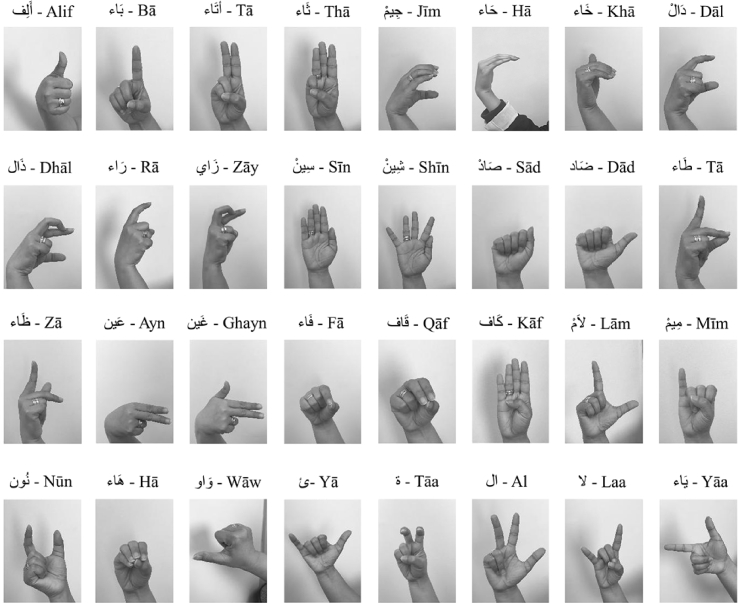
Representation of the Arabic Sign Language for Arabic Alphabets.

The ArSL2018 dataset is compiled of 54,049 images in gray scale with 64 × 64 dimension. Variations of images were introduced with different lighting and different background. Fig. 1 shows a sample of the pictures of the Arabic Sign Language signs and alphabets in the dataset. In order to assist researchers to access the ArSL2018 dataset for classification and recognition, we have collected, labelled, generated and published the ArSL2018 dataset [1]. Table 1 shows the classification of the Arabic Alphabet signs, with labels and number of images. The dataset has been identified to be sufficient for both training and classification, and has been tested as such. The dataset can be used as is and maybe increased with more variations in the second version of the dataset.

**Table 1:** Input Arabic Alphabet Sign classes with their labels and number of images.

#	Letter name in English Script	Letter name in Arabic script	# of Images	#	Letter name in English Script	Letter name in Arabic script	# of images
1	Alif	أَلِف)أ)	1672	17	Zā	ظَاء)ظ)	1723
2	Bā	بَاء) ب)	1791	18	Ayn	عَين)ع)	2114
3	Tā	أتَاء) ت)	1838	19	Ghayn	غَين)غ)	1977
4	Thā	ثَاء) ث)	1766	20	Fā	فَاء)ف)	1955
5	Jīm	جِيمْ) ج)	1552	21	Qāf	قَاف) ق)	1705
6	Hā	حَاء) ح)	1526	22	Kāf	كَاف)ك)	1774
7	Khā	خَاء) خ)	1607	23	Lām	لاَمْ)ل)	1832
8	Dāl	دَالْ) د)	1634	24	Mīm	مِيمْ)م)	1765
9	Dhāl	ذَال) ذ)	1582	25	Nūn	نُون)ن)	1819
10	Rā	رَاء) ر)	1659	26	Hā	هَاء)ه)	1592
11	Zāy	زَاي) ز)	1374	27	Wāw	وَاو)و)	1371
12	Sīn	سِينْ) س)	1638	28	Yā	يَا) ئ)	1722
13	Shīn	شِينْ) ش)	1507	29	Tāa	ة)ة)	1791
14	Sād	صَادْ)ص)	1895	30	Al	ال)ال)	1343
15	Dād	ضَاد)ض)	1670	31	Laa	ﻻ)ﻻ)	1746
16	Tā	طَاء)ط)	1816	32	Yāa	يَاء) يَاء)	1293

There are still some limitations to the ArSL2018 dataset which include, 1) dataset was collected in one location, 2) not enough lighting and noise variations were introduced, 3) the number of participants providing samples were only 40 participants. The limitations are minor and could be addressed in the second version of the dataset.

## 2 Experimental design, materials, and methods

The ArSL2018 dataset images were taken at Prince Mohammad Bin Fahd University and in the Khobar Area, Kingdom of Saudi Arabia from volunteers of different age groups. A smart Camera attached to tripod was used to capture the images. Volunteers were made to stand around 1 m away from the camera. Variations of images were introduced with different lighting, angles, timings and different background. The total number of images per alphabet varies, however, the total number of images compiled for the dataset were 54,049 images. The images were taken in RGB format with different dimensions and variations, which required pre-processing the images to make the suitable for classification and recognition. The collected images were resized to a fixed dimension 64 × 64 and converted to grayscale images, with a range of pixel values 0 to 255.
